# tDCS effect on prosocial behavior: a meta-analytic review

**DOI:** 10.1093/scan/nsab067

**Published:** 2021-06-19

**Authors:** Bo Yuan, Serenella Tolomeo, Chunliang Yang, Ying Wang, Rongjun Yu

**Affiliations:** Department of Psychology, Ningbo University, Zhejiang 315211, China; Department of Psychology, National University of Singapore 117570, Singapore; Institute of Developmental Psychology, Beijing Normal University, Beijing 100875,China; Department of Psychology, Ningbo University, Zhejiang 315211,China; Department of Management, Hong Kong Baptist University, Hong Kong 999077,China; Department of Sport, Physical Education and Health, Hong Kong Baptist University, Hong Kong 999077, China; Department of Physics, Hong Kong Baptist UniversityHong Kong 999077,China

**Keywords:** meta-analysis, prosocial behavior, transcranial direct current stimulation, stimulation parameters

## Abstract

Previous studies have shown that transcranial direct current stimulation (tDCS) could potentially promote prosocial behaviors. However, results from randomized controlled trials are inconsistent. The current meta-analysis aimed to assess the effects of anodal and cathodal tDCS using single-session protocols on prosocial behaviors in healthy young adults and explore potential moderators of these effects. The results showed that compared with sham stimulation, anodal (excitatory) stimulation significantly increased (*g* = 0.27, 95% CI [0.11, 0.43], *Z* = 3.30, *P *= 0.001) and cathodal (inhibitory) stimulation significantly decreased prosocial behaviors (*g* = −0.19, 95% CI [−0.39, −0.01], *Z* = −1.95, *P *= 0.051) using a multilevel meta-analytic model. These effects were not significantly modulated by stimulation parameters (e.g. duration, intensity and site) and types of prosocial behavior. The risk of publication bias for the included effects was minimal, and no selective reporting (e.g. *P*-hacking) was found in the *P*-curve analysis. This meta-analysis showed that both anodal and cathodal tDCS have small but significant effects on prosocial behaviors. The current study provides evidence that prosocial behaviors are linked to the activity of the ‘social brain’. Future studies are encouraged to further explore whether tDCS could effectively treat social dysfunctions in psychiatry disorders.

## Introduction

Among animals, *Homo sapiens* is unique in its capacity for widespread prosocial behavior among large and genetically heterogeneous groups of individuals. Prosocial behavior refers to a broad range of behaviors, efforts or intentions to promote or protect the well-being of other individuals, groups, organizations or societies (Penner. *et al.*, 2005; Bolino and Grant, 2016), such as helping, sharing, cooperating, trust and donating. It not only facilitates interpersonal adaptation and harmony but also enhances social welfare and social responsibility. Due to its importance and ubiquity, human prosocial behavior has received tremendous attention across scientific disciplines, including biology, economics, sociology and psychology (Thielmann *et al.*, 2020).

Prosocial behavior is a composite and multidimensional construct. It can be defined as a social behavior that benefits other people or society as a whole, such as helping, donating and cooperating (Penner. *et al.*, 2005). Several classic economic games, such as the dictator game (Forsythe *et al.*, 1994), ultimatum game (UG) (Güth *et al.*, 1982), trust game (Berg *et al.*, 1995), prisoner’s dilemma (Rapoport *et al.*, 1965) and public goods game (Samuelson, 1954), have been developed to study prosocial behavior in laboratory contexts. These game paradigms were developed to model the complexity of real-life interdependent situations in a precise yet parsimonious approach that allows assessing actual prosocial behavior in standardized experimental settings (Murnighan and Wang, 2016). In essence, economic games provide a standardized substantive model of many actual encounters and therefore have good ecological validity (Baumeister *et al.*, 2007).

Prosocial behavior involves complex cognitive and motivational processes (Coke *et al.*, 1978; Padilla-Walker and Carlo, 2014). Acting to benefit others first requires socio-cognitive abilities (e.g. the theory of mind, ToM) to understand another person’s needs and goals (Warneken, 2015). Socio-cognitive abilities enable the helping agent to realize whether and how particular actions help others to reach their goals (Frith *et al.*, 2003; Tomasello *et al.*, 2005). Prosocial behavior requires the motivation to act, which may stem from empathetic processes, and the desire to reduce the misfortune of another (De Waal, 2008; Rumble *et al.*, 2010; Xu *et al.*, 2019). Humans exhibit empathic concerns about the welfare of others and feel committed to alleviating others’ distress and pain (De Waal, 2008; Warneken, 2015).

In addition, prosocial behavior is hypothesized to engage brain regions attributed to the mentalizing and empathy brain networks (the so-called ‘social brain’) (Chakroff and Young, 2014). The right temporoparietal junction (rTPJ) is an important hub of the mentalizing network (Preckel *et al.*, 2018) and has been consistently shown in tasks that involve self-centered and other-regarding concerns (such as care about the harms, losses or feelings of others) (Soutschek *et al.*, 2016; Tang *et al.*, 2017). The rTPJ is implicated in sophisticated representations of others’ mental states and integrating them into social decisions (Lockwood *et al.*, 2019). In addition, an agent might also need to integrate cognitive and affective signals in prosocial behaviors to prospectively evaluate actions and outcomes associated with a prosocial act (Bellucci *et al.*, 2020). The ventromedial prefrontal cortex (vmPFC) has been posited to be a hub of processing action-outcome contingencies in goal-directed behaviors (Huang *et al.*, 2020). The dorsolateral prefrontal cortex (dlPFC), such as the vmPFC, further yielded functional associations with an affective domain (Bellucci *et al.*, 2020). Taken together, a set of brain regions including rTPJ, vmPFC and dlPFC, may be involved when people are engaged in prosocial behaviors.

Considerable effort has been made to promote prosocial behaviors. Previous studies have shown that transcranial direct current stimulation (tDCS) may have some effects on elevating prosocial tendency. tDCS involves the application of very low-intensity direct currents (usually ranging from 1 to 2 mA) via relatively large (25 ∼ 35 cm^2^) electrodes that are applied on the participants’ scalp above brain regions of interest for a few minutes (5 ∼ 20 min) (Bastani and Jaberzadeh, 2012; Sellaro *et al.*, 2016). The applied current causes a subthreshold modulation of the resting membrane potential of cortical neurons that alters their likelihood of ﬁring and thereby affects spontaneous cortical activity (Priori *et al.*, 1998; Nitsche *et al.*, 2008). Anodal stimulation induces depolarization of the membrane potential and increases cortical excitability, whereas cathodal stimulation does the opposite. The sham tDCS, where the current is only ramped up and down at the beginning of the stimulation to mimic skin sensations without any effective stimulation of the brain, is commonly used as a baseline condition (Ambrus *et al.*, 2012).

Changes in neural activity are not only observed during the stimulation period (online), but can also last for over 1 h after stimulation implementation (offline) if sufficient treatment (e.g. at least 9 ∼ 10 min) is implemented. The mechanism for these enduring effects is thought to be a result of long-term potentiation and long-term depression of neuronal synapses (Nitsche *et al.*, 2008). The current density (the quotient of current strength and electrode size) and stimulation duration are the two most important parameters that determine the efficacy of tDCS (Sellaro *et al.*, 2016). Both online or offline stimulation can produce significant tDCS effects on cognitive (e.g. Martin *et al.*, 2014; Hill *et al.*, 2016; Oldrati *et al.*, 2018) and motor domains (Besson *et al.*, 2019), but the sizes of effects may differ.

It has been documented that tDCS could enhance cognitive and emotional functions such as attention, memory and emotional information processing (Di Nuzzo *et al.*, 2018; Galli *et al.*, 2019). Along the same lines, recent studies provided evidence that tDCS could also alter social behaviors such as prosocial behavior. For instance, a number of studies showed that anodal *vs* sham tDCS enhanced trustworthiness (Wang *et al.*, 2016) and honesty (Maréchal *et al.*, 2017), economic (Nihonsugi *et al.*, 2015) and voluntary cooperation (Li *et al.*, 2018) and empathy to others’ pain (Wang *et al.*, 2014). Similarly, other studies reported the opposite effect of using cathodal tDCS, such as decreasing ToM, cognitive empathy (Mai *et al.*, 2016) and emotional empathy (Coll *et al.*, 2017).

While it is suggested that tDCS could potentially impact prosocial behaviors, its effectiveness needs to be quantitatively evaluated through a comprehensive meta-analysis. First, previous studies often yield inconsistent results regarding the overall effects of tDCS on the prosocial tendency. For example, a previous study found that stimulating right dorsolateral prefrontal cortex (rDLPFC) by tDCS produced different effects on voluntary and sanction-based social norm compliance (Ruff *et al.*, 2013). Another study reported that the application of tDCS over the prefrontal cortex enhanced the trustee’s repayment through altruism (Zheng *et al.*, 2016), whereas no such significant effect was reported on interpersonal trust as the trustor (Zheng *et al.*, 2017). There is large heterogeneity in experimental prosocial tasks due to the wide range of prosocial behaviors such as trust, trustworthiness, altruism and pain empathy. The tDCS effects on prosociality may be limited to certain social behaviors, but not others. Second, similar to other research domains, tDCS research suffers from replication risk, *P*-hacking (file-drawer), publication bias, small sample size and hypothesizing after the results are known (problems HARKing) (Simmons *et al.*, 2011), casting doubt on the efficacy of tDCS and the replicability of tDCS effects. A quantitative assessment of the risk of publication bias and selective reporting (e.g. *P*-hacking) in this field is called for. Third, substantial research design variations exist across studies in terms of stimulation parameters and protocols, leading to inconsistent research findings (Galli *et al.*, 2019). It is well-known that a variety of factors, besides the polarity of stimulation, may modulate the magnitude of the tDCS effects, such as electrode placement and size, current density, intensity and duration of stimulation and motivational factors (Sellaro *et al.*, 2016). Hence, it is necessary to assess these variables’ potential moderating roles in the tDCS effects on prosocial behavior in a comprehensive meta-analysis. Finally, researchers have stimulated different parts of the ‘social brain’, including dlPFC, rTPJ and vmPFC. Although these regions are involved in social cognition, it is unclear whether the tDCS effect is stronger on one site than the other, which needs to be unraveled by a meta-analysis.

To the best of our knowledge, no previous meta-analysis has examined the effects of tDCS on prosocial behavior. In the present study, two meta-analyses were conducted to assess the effects of anodal and cathodal tDCS stimulations on prosocial behavior. Potential moderators of tDSC effects, such as stimulation site and types of prosocial behavior, were tested in the sub-group meta-analyses. Further meta-regression analyses were implemented to examine whether the magnitude of tDCS effects varied as a function of specific stimulation parameters (such as current density and stimulation duration). Finally, the *p*-curve analysis was also conducted to assess the evidential value of these effects. To sum up, the purpose of this systematic review and meta-analysis was to analyze the effect of tDCS on prosocial behaviors and explore potential moderators of such an effect in healthy adults.

## Method

### Literature search

We conducted searches for published and unpublished articles/reports in English language in the following databases: Web of Science, Science Direct, PubMed and Google Scholar. The search terms included [‘Transcranial Direct Current Stimulation’ OR ‘tDCS’] AND [‘trust’ OR ‘cooperation’ OR ‘prosocial behavior’ OR ‘helping behavior’ OR ‘altruism’ OR ‘honesty’ OR ‘altruism behavior’ OR ‘empathy’]. In addition, a few review articles and their reference lists were screened (Boggio *et al.*, 2016; Sellaro *et al.*, 2016; Di Nuzzo *et al.*, 2018). This work followed the Preferred Reporting Items for Systematic Reviews and Meta-Analyses (PRISMA) guidelines (Moher *et al.*, 2015). Two reviewers (WY and YB) independently screened the titles and abstracts of articles identified in the initial search strategy against the inclusion criteria (see below) and potentially relevant studies were retrieved for full-text screening. Discrepancies between reviewers were settled through a discussion.

### Inclusion and exclusion criteria

The following inclusion criteria were implemented: (i) only manuscripts written in English and available before December 2020 were considered; (ii) only studies that involved randomized, sham-controlled trials were included; (iii) participants have to be healthy population; (iv) the main outcome of the study has to be a measure of prosocial behavior such as trust, trustworthiness, altruism, honesty, empathy and ToM^1^; (v) only studies that provided sufficient data [e.g. *M* standard deviation (s.d.)*, t, F*] for effect size calculation were included and (vi) only studies implemented anodal and cathodal stimulation in any brain region and any type of electrode were considered.

### Data extraction

For each study, we extracted means and standard deviations of the outcome measures of interest, along with the sample sizes. The same two reviewers independently extracted data using a data extraction form. The following variables were extracted according to a structured checklist previously elaborated by the authors: (i) metadata (i.e. authorship, publication date, journal); (ii) demographics (sample size in each group, mean age and gender); (iii) prosocial behavior types such as trust, trustworthiness, altruism (e.g. altruistic giving or help), honesty, empathy, ToM; (iv) stimulation sites such as rDLPFC, vmPFC, rTPJ, lDLPFC (left dorsolateral prefrontal cortex), rOFC (right orbitofrontal cortex) and SI (somatosensory cortex) and (v) characteristics of the tDCS technique (intensities of the current, stimulation durations and online/offline stimulation).

Wherein mean and s.d. values were not provided for anodal/cathodal and sham condition as numerical data, they were pooled out from the graphs with Plot Digitizer software (Jelicic Kadic *et al.*, 2016). Some of the studies included in the current meta-analysis tested multiple experimental variables within-subjects or involved other types of non-independent statistical comparisons. We treated stimulated brain areas, prosocial behavior types, characteristics of the tDCS technique as independent data. We were aware that computing different effect sizes for the same or overlapping sets of participants and treating them as completely unrelated effect sizes violate the assumptions of the traditional meta-analytic method. However, the variables mentioned above were of primary interest and were included as moderators; therefore, we reasoned that data reduction would have resulted in a loss of relevant information. To address this, we also reported the results fitted a two-level model with random effects at the study level, using the rma.mv function of the ‘metaphor’ R package (Viechtbauer, 2010). This strategy allowed us to control for dependencies in the dataset, while preserving the information conveyed by each effect size (Galli *et al.*, 2019).

### Statistical approach and publication bias

For the main outcome, the standardized mean difference and the pooled s.d. for each comparison were calculated. The Hedges’ *g* was used as the measure of effect sizes, which is appropriate for studies with small sample sizes. All meta-analyses were conducted using random-effects models. Heterogeneity was evaluated with *I*^2^ and χ^2^ tests (Higgins and Thompson, 2002). Publication bias was examined by Egger’s regression test and a funnel plot. The Duval and Tweedie ‘Trim and Fill’ procedure (Duval and Tweedie, 2000) was implemented to adjust for any suspected publication bias using a random-effects model.

Further analyses were performed to explore the potential moderators such as age, the current density of stimulation, duration of stimulation, stimulation sites and prosocial behavior types. Current density (A/m^2^) was estimated by dividing the electric current (Amperes, A) by the electrode surface area (square meters, m^2^).

### Quality assessment

A quality assessment was conducted for each included study by using the Physiotherapy Evidence Database (PEDro scale) (Maher *et al.*, 2003) to assess the methodological quality of included articles (Supplementary Table S1). The PEDro scale includes 11 specific criteria, graded on a ‘yes’/‘no’ scale in which the first item relates to external validity and the other 10 items assess the internal validity of a clinical trial (Bastani and Jaberzadeh, 2012). The first criterion does not count toward the overall score that the article receives for the quality of its study design. The PEDro scale is marked out of 10, and a higher PEDro score represents a higher assumed ‘quality’ of the trial.

### P-Curve analysis

Simonsohn *et al.* (2014) proposed a method for diagnosing *P*-hacking by considering the distribution of significant *P*-values obtained over a series of independent studies. *P*-curve analysis assesses the distribution of *P*-values among published articles to diagnose whether the findings provide evidence for a true phenomenon, or whether they likely reflect an artifact of publication bias and *P*-hacking. The logic is that studies demonstrating true effects (where the null is false) will be more likely to produce particularly low *P* values (*Ps* < 0.025) than those in the higher range of significance (0.025 < *Ps* < 0.05). The distribution of *P* values for a true effect should thus be right-skewed. Studies that investigate null effects produce an equal distribution of *P* values, resulting in a uniform *P* curve. This type of ‘flat’ *P* curve suggests that the body of literature lacks evidentiary value (Shariff *et al.*, 2016). We conducted *P*-curve analyses using *P* values of the main effects included in the meta-analyses to assess their evidential value (Köbis *et al.*, 2019).

## Results

### Study characteristics

**Fig. 1.**
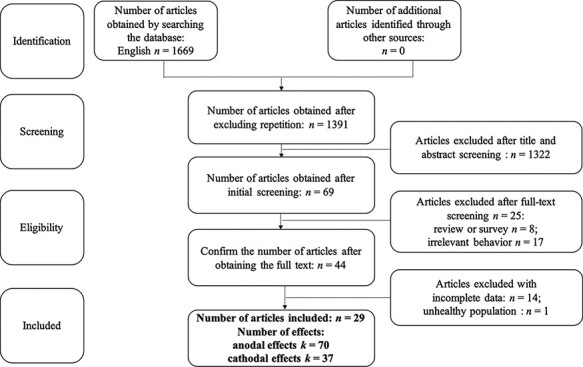
Flowchart of study selection for our systematic review and meta-analysis.

The initial literature search returned 1,669 articles, of which 278 were duplicates and 1,322 were excluded for not meeting the inclusion criteria (Figure 1 for more details). Of the remaining 69 studies, 44 with prosocial behavior outcome measures were identified as appropriate for inclusion in this review. From these 44 studies, 14 were excluded due to missing information for effect size calculation, and one was removed because of involving dementia participants. There were a total of 29 studies identified for the current meta-analysis, including 70 effect sizes for the anodal tDCS effect and 38 effect sizes for the cathodal tDCS effect. Among these studies, the common stimulation sites are vmPFC, dlPFC and rTPJ (Supplementary Figure S1) and the typical experimental paradigms used include trust game, ultimate (dictator) game, public good game and empathy task (Supplementary Figure S2).

### Overall meta-analysis

**Fig. 2.**
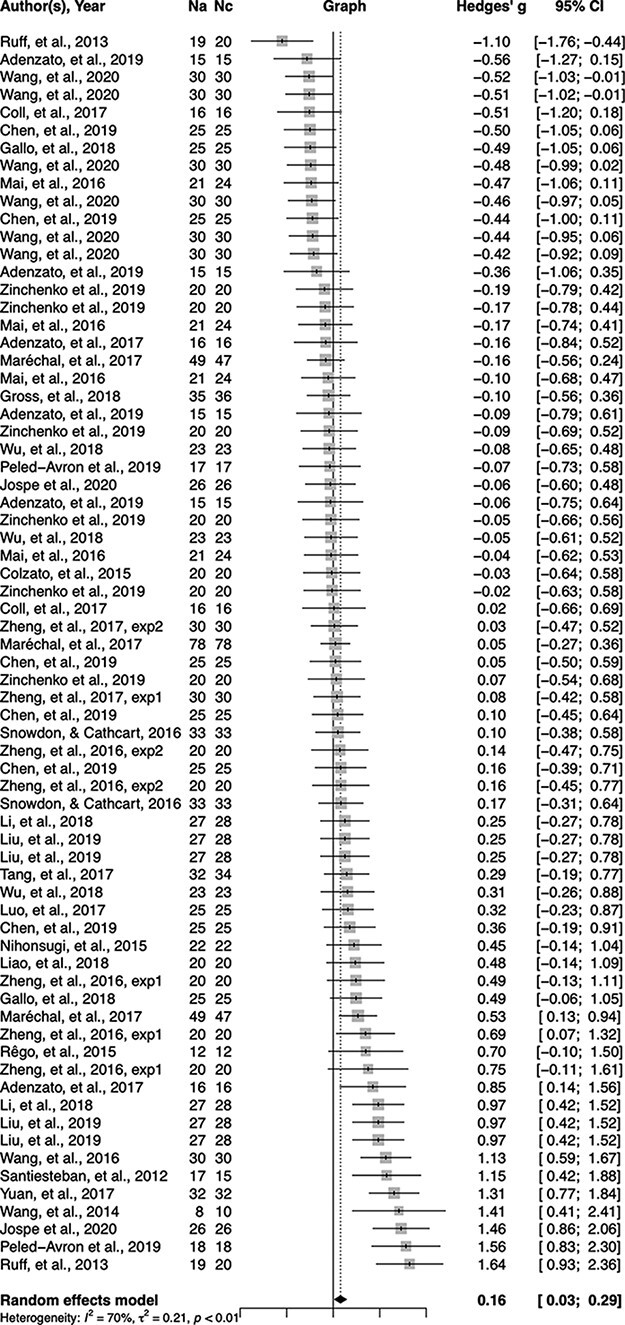
Forest plot of anodal tDCS effect sizes (Hedges’ *g*) and 95% CI for each study and the overall effect size.

**Fig. 3.**
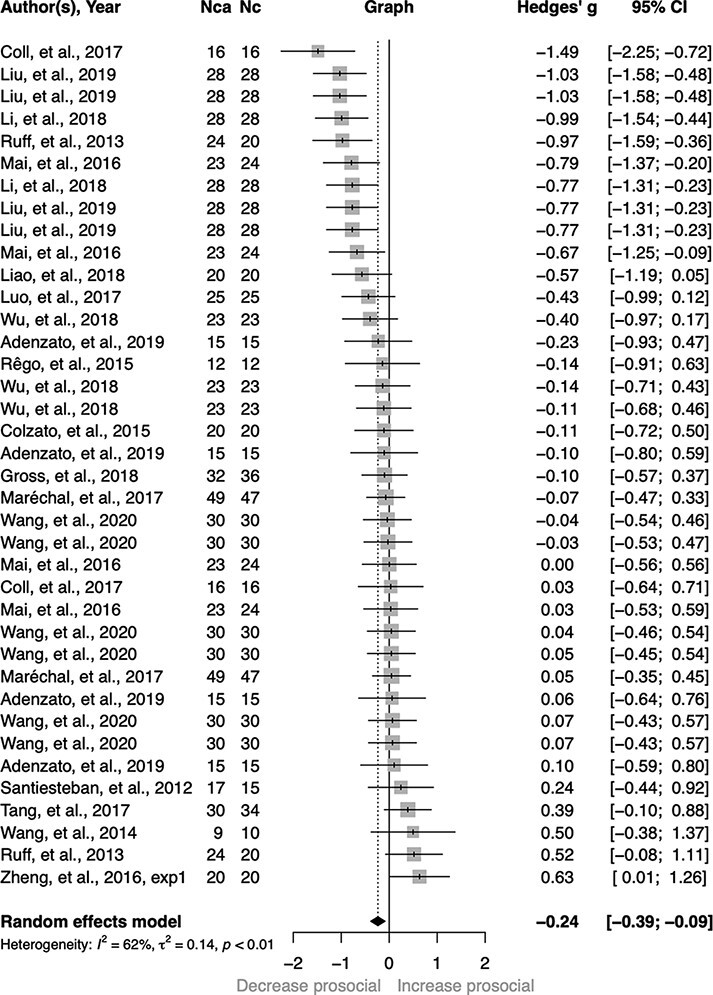
Forest plot of cathodal tDCS effect sizes (Hedges’ *g*) and 95% CI for each study and the overall effect size.

Tables 1 and 2 list the anodal/cathodal tDCS effects on prosocial behaviors and the corresponding study characteristics. We found that anodal tDCS, in comparison with sham tDCS, enhanced prosocial behavior to a modest extent, *g* = 0.16, 95% CI [0.03, 0.29], *Z* = 2.44, *P *= 0.015 (Figure 2). The anodal tDCS effect was still significant when we fitted a two-level model with random effects at the study level, *g* = 0.27, 95% CI [0.11, 0.43], *Z* = 3.30, *P *= 0.001. Cathodal tDCS, compared with sham stimulation, modestly decreased prosocial behaviors, *g* = −0.24, 95% CI [−0.39, −0.09], *Z* = −3.08, *P *= 0.002 (Figure 3). This effect was still significant when we fitted a two-level model with random effects at the study level, *g* = −0.19, 95% CI [−0.39, −0.01], *Z* = −1.95, *P *= 0.051.

**Table 1.** Overview of the studies included in the meta-analysis of anodal tDCS effect

Author, year, Experiment	Age (years)	*N*(a)	*N*(c)	Active brain	Prosocial behavior	Duration (min)	Area (cm^2^)	Intensity (mA)	Q_Score	Hedges’ *g*
Adenzato *et al*. (2017)	23.50	16	16	vmPFC	ToM	6	35	1.00	6	0.85
Adenzato *et al*. (2017)	23.50	16	16	vmPFC	ToM	6	35	1.00	6	−0.16
Adenzato *et al*. (2019)	68.3	15	15	vmPFC	ToM	6	35	1.5	8	−0.06
Adenzato *et al*. (2019)	68.3	15	15	vmPFC	ToM	6	35	1.5	8	−0.09
Adenzato *et al*. (2019)	67.5	15	15	vmPFC	ToM	6	35	1.5	8	−0.36
Adenzato *et al*. (2019)	67.5	15	15	vmPFC	ToM	6	35	1.5	8	−0.56
Chen *et al*. (2019)	20.5	25	25	rDLPFC	Altruism	30	35	1.5	7	−0.44
Chen *et al*. (2019)	20.5	25	25	rDLPFC	Altruism	30	35	1.5	7	0.16
Chen *et al*. (2019)	20.5	25	25	rDLPFC	Altruism	30	35	1.5	7	−0.5
Chen *et al*. (2019)	20.5	25	25	rDLPFC	Altruism	30	35	1.5	7	0.36
Chen *et al*. (2019)	20.5	25	25	rDLPFC	Altruism	30	35	1.5	7	0.05
Chen *et al*. (2019)	20.5	25	25	rDLPFC	Altruism	30	35	1.5	7	0.1
Coll *et al.* (2017)	26.54	16	16	rTPJ	Empathy	20	35	2.00	7	−0.51
Coll *et al.* (2017)	25.69	16	16	rTPJ	Empathy	20	35	2.00	7	0.02
Colzato *et al*. (2015)	21.00	20	20	vmPFC	Trust	20	35	1.00	9	−0.03
Gallo *et al*. (2018)	24.50	25	25	SI	Empathy	18	35	1.50	7	0.49
Gallo *et al*. (2018)	24.50	25	25	SI	Empathy	18	35	1.50	7	−0.49
Gross *et al*. (2018)	21.40	35	36	rDLPFC	Altruism	30	35	2.00	9	−0.10
Jospe *et al*. (2020)	23.94	26	26	rIFG	Empathy	15	16	1.25	7	−0.06
Jospe *et al*. (2020)	23.94	26	26	rIFG	Empathy	15	16	1.25	7	1.46
Li *et al.* (2018)	24.04	27	28	rDLPFC	Altruism	15	35	1.00	8	0.97
Li *et al.* (2018)	24.04	27	28	rDLPFC	Altruism	15	35	1.00	8	0.25
Liao *et al*. (2018)	20.80	20	20	vmPFC	Altruism	20	25	2.00	6	0.48
Liu *et al*. (2019)	25.6	27	28	rDLPFC	Altruism	15	35	1.5	7	0.97
Liu *et al*. (2019)	25.6	27	28	rDLPFC	Altruism	15	35	1.5	7	0.25
Liu *et al*. (2019)	25.6	27	28	rDLPFC	Altruism	15	35	1.5	7	0.97
Liu *et al*. (2019)	25.6	27	28	rDLPFC	Altruism	15	35	1.5	7	0.25
Luo *et al*. (2017)	19.80	25	25	rDLPFC	Altruism	20	35	2.00	8	0.32
Mai *et al.* (2016)	22.80	21	24	rTPJ	Empathy	20	35	1.50	8	−0.47
Mai *et al.* (2016	22.80	21	24	rTPJ	ToM	20	35	1.50	8	−0.10
Mai *et al.* (2016)	22.80	21	24	rTPJ	Empathy	20	35	1.50	8	−0.04
Mai *et al.* (2016)	22.80	21	24	rTPJ	ToM	20	35	1.50	8	−0.17
Maréchal *et al.* (2017)	23.00	49	47	rDLPFC	Honesty	30	35	1.50	7	0.53
Maréchal *et al.* (2017)	23.00	49	47	rDLPFC	Altruism	30	35	1.50	7	−0.16
Maréchal *et al.* (2017)	23.00	78	78	rDLPFC	Altruism	30	35	1.50	7	0.05
Nihonsugi *et al.* (2015)	20.50	22	22	rDLPFC	Trust	9	35	2.00	6	0.45
Peled-Avron *et al*. (2019)	25.2	17	17	rIFG	Empathy	15	25	1.5	7	−0.07
Peled-Avron *et al*. (2019)	25.2	18	18	rIFG	Empathy	15	25	1.5	7	1.56
Rêgo *et al*. (2015)	24.00	12	12	rDLPFC	Empathy	15	35	2.00	8	0.70
Ruff *et al.* (2013)	22.00	19	20	rDLPFC	Altruism	12	35	1.00	8	1.64
Ruff *et al.* (2013)	22.00	19	20	rDLPFC	Altruism	12	35	1.00	8	−1.10
Santiesteban *et al.* (2012)	26.50	17	15	rTPJ	ToM	20	35	1.00	5	1.15
Snowdon *et al*. (2016)	23.07	33	33	rDLPFC	Empathy	20	35	1.50	8	0.10
Snowdon *et al*. (2016)	23.07	33	33	rDLPFC	Altruism	20	35	1.50	8	0.17
Tang *et al.* (2017)	22.36	32	34	rTPJ	Altruism	20	35	1.50	8	0.29
Wang *et al.* (2014)	23.60	8	10	lDLPFC	Empathy	5	35	2.00	7	1.41
Wang *et al.* (2016)	22.37	30	30	rOFC	Trust	15	9	2.00	7	1.13
Wang *et al*. (2020)	22.35	30	30	vmPFC	Altruism	20	35	1	7	−0.51
Wang *et al*. (2020)	22.35	30	30	vmPFC	Altruism	20	35	1	7	−0.52
Wang *et al*. (2020)	22.35	30	30	vmPFC	Altruism	20	35	1	7	−0.48
Wang *et al*. (2020)	23.35	30	30	vmPFC	Altruism	20	35	1	7	−0.44
Wang *et al*. (2020)	23.35	30	30	vmPFC	Altruism	20	35	1	7	−0.46
Wang *et al*. (2020)	23.35	30	30	vmPFC	Altruism	20	35	1	7	−0.42
Wu *et al*. (2018)	24.39	23	23	rIFG	Empathy	20	35	1.5	7	0.31
Wu *et al*. (2018)	24.39	23	23	rIFG	Empathy	20	35	1.5	7	−0.05
Wu *et al*. (2018)	24.39	23	23	rIFG	Empathy	20	35	1.5	7	−0.08
Yuan *et al*. (2017)	23.57	32	32	vmPFC	Empathy	30	35	1.50	8	1.31
Zheng *et al.* (2016), exp1	21.50	20	20	vmPFC	Trust	20	35	2.00	8	0.49
Zheng *et al.* (2016), exp1	21.50	20	20	vmPFC	Trustworthiness	20	35	2.00	8	0.75
Zheng *et al.* (2016), exp1	21.50	20	20	vmPFC	Altruism	20	35	2.00	8	0.69
Zheng *et al.* (2016), exp2	21.50	20	20	rDLPFC	Trustworthiness	20	35	2.00	8	0.16
Zheng *et al.* (2016), exp2	21.50	20	20	rDLPFC	Altruism	20	35	2.00	8	0.14
Zheng *et al.* (2017), exp1	21.00	30	30	rDLPFC	Trust	20	35	2.00	8	0.08
Zheng *et al.* (2017), exp2	21.00	30	30	rDLPFC	Trust	20	35	2.00	8	0.03
Zinchenko *et al*. (2019)	21.5	20	20	rTPJ	Altruism	15	25	1.5	7	−0.19
Zinchenko *et al*. (2019)	21.5	20	20	rDLPFC	Altruism	15	25	1.5	7	−0.05
Zinchenko *et al*. (2019)	21.5	20	20	rTPJ	Altruism	15	25	1.5	7	−0.09
Zinchenko *et al*. (2019)	21.5	20	20	rDLPFC	Altruism	15	25	1.5	7	0.07
Zinchenko *et al*. (2019)	21.5	20	20	rTPJ	Altruism	15	25	1.5	7	−0.17
Zinchenko *et al*. (2019)	21.5	20	20	rDLPFC	Altruism	15	25	1.5	7	−0.02

*Note*: Q_Score = quality score; *N*(a) = sample size of anodal condition; *N*(c) = sample size of control (sham) condition; ToM = theory of mind; vmPFC = ventromedial prefrontal cortex; rDLPFC = right dorsolateral prefrontal cortex; lDLPFC = left dorsolateral prefrontal cortex; rTPJ = right temporoparietal junction; rOFC = right orbitofrontal cortex; SI = somatosensory cortex.

**Table 2.** Overview of the studies included in the meta-analysis of cathode tDCS effect

Author, year, Exp	Age (years)	*N*(ca)	*N*(c)	Deactivate brain	Prosocial behavior	Duration (min)	Area (cm^2^)	Intensity (mA)	Q_Score	Hedges’ *g*
Adenzato *et al*. (2019)	68.3	15	15	vmPFC	ToM	6	35	1.5	8	−0.23
Adenzato *et al*. (2019)	68.3	15	15	vmPFC	ToM	6	35	1.5	8	0.1
Adenzato *et al*. (2019)	67.5	15	15	vmPFC	ToM	6	35	1.5	8	0.06
Adenzato *et al*. (2019)	67.5	15	15	vmPFC	ToM	6	35	1.5	8	−0.1
Coll *et al.* (2017)	26.54	16	16	rTPJ	Empathy	20	35	2.00	7	−1.49
Coll *et al.* (2017)	25.69	16	16	rTPJ	Empathy	20	35	2.00	7	0.03
Colzato *et al*. (2015)	21.00	20	20	vmPFC	Trust	20	35	1.00	9	−0.11
Gross *et al*. (2018)	21.40	32	36	rDLPFC	Altruism	30	35	2.00	9	−0.10
Li *et al.* (2018)	24.04	28	28	rDLPFC	Altruism	15	35	1.00	8	−0.77
Li *et al.* (2018)	24.04	28	28	rDLPFC	Altruism	15	35	1.00	8	−0.99
Liao *et al*. (2018)	20.80	20	20	vmPFC	Altruism	20	25	2.00	6	−0.57
Liu *et al*. (2019)	25.6	28	28	rDLPFC	Altruism	15	35	1.5	7	−0.77
Liu *et al*. (2019)	25.6	28	28	rDLPFC	Altruism	15	35	1.5	7	−1.03
Liu *et al*. (2019)	25.6	28	28	rDLPFC	Altruism	15	35	1.5	7	−0.77
Liu *et al*. (2019)	25.6	28	28	rDLPFC	Altruism	15	35	1.5	7	−1.03
Luo *et al*. (2017)	19.80	25	25	rDLPFC	Altruism	20	35	2.00	8	−0.43
Mai *et al.* (2016)	22.80	23	24	rTPJ	Empathy	20	35	1.50	8	−0.79
Mai *et al.* (2016)	22.80	23	24	rTPJ	ToM	20	35	1.50	8	−0.67
Mai *et al.* (2016)	22.80	23	24	rTPJ	Empathy	20	35	1.50	8	0.00
Mai *et al.* (2016)	22.80	23	24	rTPJ	ToM	20	35	1.50	8	0.03
Maréchal *et al.* (2017)	23.00	49	47	rDLPFC	Honesty	30	35	1.50	7	0.05
Maréchal *et al.* (2017)	23.00	49	47	rDLPFC	Altruism	30	35	1.50	7	−0.07
Rêgo *et al*. (2015)	24.00	12	12	rDLPFC	Empathy	15	35	2.00	8	−0.14
Ruff *et al.* (2013)	22.00	24	20	rDLPFC	Altruism	12	35	1.00	8	−0.97
Ruff *et al.* (2013)	22.00	24	20	rDLPFC	Altruism	12	35	1.00	8	0.52
Santiesteban *et al.* (2012)	26.50	17	15	rTPJ	ToM	20	35	1.00	5	0.24
Tang *et al.* (2017)	22.36	30	34	rTPJ	Altruism	20	35	1.50	8	0.39
Wang *et al.* (2014)	23.60	9	10	lDLPFC	Empathy	5	35	2.00	7	0.50
Wang *et al*. (2020)	22.35	30	30	vmPFC	Altruism	20	35	1	7	−0.04
Wang *et al*. (2020)	22.35	30	30	vmPFC	Altruism	20	35	1	7	0.04
Wang *et al*. (2020)	22.35	30	30	vmPFC	Altruism	20	35	1	7	0.07
Wang *et al*. (2020)	23.35	30	30	vmPFC	Altruism	20	35	1	7	−0.03
Wang *et al*. (2020)	23.35	30	30	vmPFC	Altruism	20	35	1	7	0.05
Wang *et al*. (2020)	23.35	30	30	vmPFC	Altruism	20	35	1	7	0.07
Wu *et al*. (2018)	24.39	23	23	rIFG	Empathy	20	35	1.5	7	−0.4
Wu *et al*. (2018)	24.39	23	23	rIFG	Empathy	20	35	1.5	7	−0.14
Wu *et al*. (2018)	24.39	23	23	rIFG	Empathy	20	35	1.5	7	−0.11
Zheng *et al.* (2016), exp1	21.50	20	20	vmPFC	Trustworthiness	20	35	2.00	8	0.63

*Note*: Q_Score = quality score; *N*(ca) = sample size of cathodal condition; *N*(c) = sample size of control (sham) condition; ToM = theory of mind; vmPFC = ventromedial prefrontal cortex; rDLPFC = right dorsolateral prefrontal cortex; lDLPFC = left dorsolateral prefrontal cortex; rTPJ = right temporoparietal junction.

In addition, we analyzed the anodal and cathodal tDCS effects on prosocial behaviors after removing the empathy and ToM items in the meta-analysis. Empathy and ToM are important foundations for prosocial behavior, but may not be considered as forms of prosocial acts. Nevertheless, anodal tDCS still enhanced prosocial behavior (*k* = 44) to a small extent, *g* = 0.13, 95% CI [−0.02, 0.27], *Z* = 1.73, *P *= 0.082, and this effect was significant when we fitted a two-level model with random effects at the study level, *g* = 0.21, 95% CI [0.03, 0.39], *Z* = 2.33, *P *= 0.020. In addition, cathodal tDCS significantly decreased prosocial behaviors after removing empathy and ToM effects (*k* = 22), *g* = −0.26, 95% CI [−0.47, −0.05], *Z* = −2.42, *P *= 0.015. However, the effect was not significant using two-level model with random effects: *g* = −0.21, 95% CI [−0.49, 0.07], *Z* = −1.48, *P *= 0.140.

### Heterogeneity test and publication bias detection

**Fig. 4.**
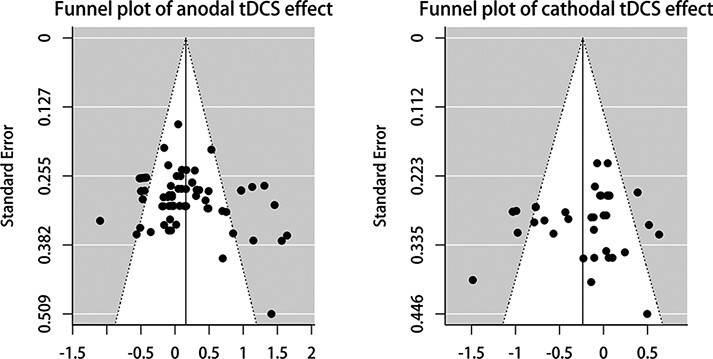
Funnel plots representative of publishing bias of two meta-analyses.

The *Q* test for heterogeneity was signiﬁcant in our two meta-analysis [*Q*(69) = 232.17, *P *< 0.001*, I*^2^ = 72.00%; *Q*(37) = 96.23, *P *< 0.001*, I*^2^ = 62.38%], indicating the necessity for exploring potential moderators of these effects (Borenstein *et al.*, 2011). To assess the potential publication bias, we first examined the adjusted effect size estimates following Duval and Tweedie’s (2000) Trim-and-Fill procedure. No missing effects were detected by the Trim-and-Fill method (Figure 4). Similarly, the Egger’s regression tests indicate that the risk of publication bias in both meta-analyses was little (the anodal effects: *Z* = 1.29, *P* = 0.194; the cathodal effects: *Z* = −1.22, *P* = 0.222).

### Moderator analyses

#### Categorical variables.

For the anodal tDCS effects, moderator analyses (sub-group analyses) revealed no main effects of the types of prosocial behavior (*Q*_B_(3) = 3.56, *P *= 0.313), active brain areas (*Q*_B_(2) = 3.31, *P *= 0.191) as well as online/offline stimulation (*Q*_B_(1) <0.001, *P *= 0.976). Along the same lines, the types of social behavior (*Q*_B_(2) = 1.83, *P *= 0.400), active brain areas (*Q*_B_(1) = 0.89, *P *= 0.347) and online/offline stimulation (*Q*_B_(1) = 1.38, *P *= 0.241) did not significantly moderate the cathodal tDCS effects. Note that those levels with the number of effects (*k*) less than 5 were excluded in the above moderator analyses, given that a small number of effects (*k* < 5) might result in low statistical power and be unable to produce reliable results.

#### Continuous variables.

**Fig. 5.**
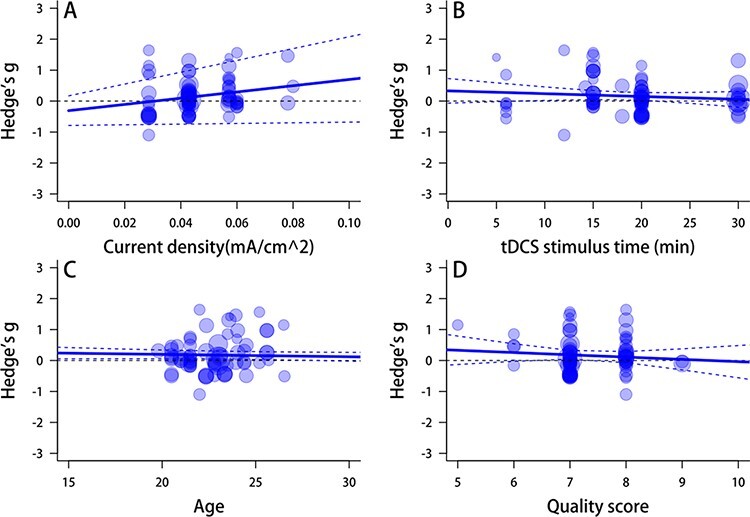
Meta-regression of anodal tDCS effect.

**Fig. 6.**
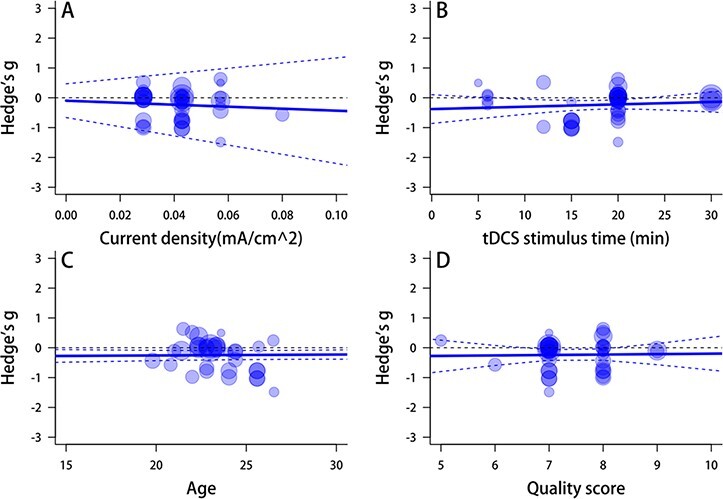
Meta-regression of cathodal tDCS effect.

Meta-regression analyses evidenced that only current density significantly moderated the anodal effects tDCS on prosocial behaviors (*Q*_B_(1) = 3.39, *P *= 0.047). No modulating roles of other various continuous variables in the anodal effects tDCS on prosocial behaviors such as stimulating duration (*Q*_B_(1) = 0.80, *P *= 0.371), age (*Q*_B_(1) = 1.40, *P *= 0.237) and quality score (*Q*_B_(1) = 0.581, *P *= 0.446) (Figure 5) were found. These factors also did not significantly moderate the cathodal tDCS effects: current density, *Q*_B_(1) = 0.25, *P *= 0.617; stimulate duration, *Q*_B_(1) = 0.38, *P *= 0.538; age, *Q*_B_(1) = 0.25, *P *= 0.615 and quality score, *Q*_B_(1) = 0.02, *P *= 0.889 (Figure 6).

#### P-Curve analysis.

**Fig. 7.**
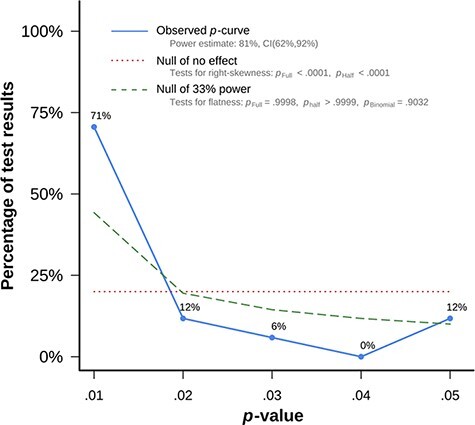
Observed *P*-curve for anodal tDCS effects on prosocial behavior in the meta-analysis. The observed *P*-curve includes 17 statistically significant (*P* < 0.05) results, of which 14 were *P* < 0.025. Fifty-three additional results were entered but excluded from the *P*-curve because they were *P* > 0.05. The blue line shows the observed *P*-curve, the dashed red line shows the uniform distribution of the *P*-values and the green line plots the right-skewed distribution for a power level of 33%.

*P*-curve analysis combines the half and full *P*-curve to make inferences about evidential value. In particular, if the half *P*-curve test is right-skewed with *P *< 0.05 or both the half and full tests are right-skewed with *P *< 0.1, then *P*-curve analysis indicates the presence of evidential value (Simonsohn *et al.*, 2015). Our *P*-curve analysis revealed that it was significantly right-skewed for the anodal tDCS effects, Full *P*-curve: *z *= −6.78, *P *< 0.001; Half *P*-curve: *z *= −6.88, *P *< 0.001 (Figure 7); and the cathodal tDCS effects, Full *P*-curve: *z *= −4.68, *P *< 0.001; Half *P*-curve: *z *= −3.90, *P *< 0.001, suggesting sufficient evidence for justifying the existence of the anodal and cathodal effects on prosocial behaviors.

Similarly, *P*-curve analysis indicates that evidential value is inadequate or absent if the 33% power test is *P *< 0.05 for the full *P*-curve or both the half *P*-curve and binomial 33% power test are *P *< 0.1. The flatter than 33% power test in the current meta-analysis is non-significant binomial test: *Pp*Binomial* *= 0.903, Full *P*-curve: *z *= 3.54, *P *> 0.999, Half *P*-curve: *z *= 6.774, *P *> 0.999 in the anodal tDCS effects (*P*Binomial* *= 0.97, Full *P*-curve: *z *= 2.13, *P *= 0.983, Half *P*-curve: *z *= 4.430, *P *> 0.999 in the cathodal tDCS effects), indicating that evidential value is adequate to support the existence of the effects. These results suggest that the included studies reflect a real effect of the relationship between anodal (cathodal) tDCS and prosocial behavior, rather than publication bias or *P*-hacking.

## Discussion

The current meta-analyses found that anodal tDCS promoted prosocial behaviors, whereas cathodal tDCS inhibited them. The risk of publication bias for the included effect sizes was low. These effects were not modulated by a range of factors such as stimulation site, types of prosocial behavior and stimulation parameters (e.g. stimulate duration, current intensity).^2^ The *P*-curve analysis showed that the *P*-values for anodal and cathodal tDCS effects were significantly right-skewed, indicating evidential value supporting the existence of the anodal and cathodal tDCS effects on prosocial behaviors.

tDCS technique was proved to be able to alter many aspects of cognitive processes and behaviors (such as enhancing perceptual and motor learning) among healthy adults (Falcone *et al.*, 2012; Galli *et al.*, 2019). However, the impact of tDCS on social decision-making is often debated (Sellaro *et al.*, 2016). The current meta-analysis indicated that anodal tDCS increases prosocial behaviors and cathode tDCS reduces prosocial behaviors. Such anodal-excitation and cathodal-inhibition dual-polarity effect have not been consistently observed in previous tDCS studies. For example, a number of studies have reported the lack of inhibitory cathodal effects on perception and motor learning, indicating that cathodal stimulation effects are in general less reliable in modulating cognitive processes (Jacobson *et al.*, 2012). In the social domain, several studies also reported a lack of cathodal effects, but significant anodal effects (Kuehne *et al.*, 2015; Sellaro *et al.*, 2015). Similarly, in the current meta-analysis, several studies reported significant anodal effects but non-significant cathodal effects (e.g. Santiesteban *et al.*, 2012; Maréchal *et al.*, 2017). However, lumping together these studies, we found that weighted mean effect sizes of the anodal and cathodal stimulations were generally comparable, although the cathodal effects tended to be slightly weaker than the anodal effects when we fitted a two-level model with random effects at the study level. The bidirectional effects of tDCS on prosociality may suggest that the initial neuronal activation state in the ‘social brain’ is subject to substantial modulation.

We also found that the risk of publication bias of the current meta-analyses was low. In addition, the *P*-curve analysis indicated that anodal or cathodal tDCS had a real effect on prosocial behaviors. Importantly, the effect sizes of anodal tDCS (*g* = 0.27) and cathode tDCS (*g* = −0.19) are relatively small, and the confidence interval range was relatively wide, with the lower limit close to zero. Hence, overall, the tDCS effects on prosocial behavior are relatively weak, and further RCTs with larger sample sizes are warranted. These findings also suggest that the observed tDCS effects on prosocial behaviors are unlikely to be driven by publication bias and *P*-hacking, as shown by the above *P*-curve analyses.

Although the results revealed that the included effects were substantially heterogeneous, no reliable significant moderators were found. Subgroup analyses indicated that neither the types of social behavior nor active brain areas significantly moderate the effects. In the identified literature, prosocial behavior mainly included the following categories: trust, trustworthiness, altruism, honesty, empathy and ToM. Our results showed that the tDCS effect did not significantly differ across those types of prosocial behaviors, indicating that tDCS stimulation has a general effect on prosociality independent of specific social tasks or domains. However, it is worth noting that the non-significant effects for the sub-types of social behavior may result from the small number of effect sizes in each category. Despite our results did not show significant moderate effects, several studies included in our meta-analysis reported that the application of anodal tDCS over the prefrontal cortex enhanced the trustee’s repayment through altruism (Wang *et al.*, 2016; Zheng *et al.*, 2016), whereas no such significant effect was reported on investment as the trustor (Zheng *et al.*, 2017). Future studies should further explore prosocial behaviors that are most sensitive to tDCS manipulation using more rigorous procedures that consider factors known to influence tDCS.

In the included studies in our meta-analysis, the commonly used stimulation brain areas are vmPFC, rDLPFC and rTPJ. These regions are part of the ‘social brain’ circuits (Adolphs, 2003, 2009), which are involved in the process of metalizing and empathy (Chakroff and Young, 2014). It has been demonstrated that the vmPFC is associated with decisions involving trustworthiness and altruism (Waytz *et al.*, 2012). For example, patients with lesions in the vmPFC showed less trustworthiness and altruism than control subjects (Moretto *et al.*, 2013). In addition, clinical lesion studies reported that patients with damage to the vmPFC gave significantly less allocation in the dictator game as well as showed less trustworthiness in the trust game. The vmPFC has been posited to be a hub of processing action-outcome contingencies in goal-directed behaviors (Huang *et al.*, 2020), which might indicate that the vmPFC is indispensable in both altruistic and trustworthy decisions (Krajbich *et al.*, 2009). In addition, rDLPFC has been shown to play an important role in social norm compliance. For instance, Sanfey *et al.* (2003) showed that dlPFC was associated with social norm compliance in the UG. Similarly, Ruff *et al.* (2013) reported that social norm compliance was changed while the activity of rDLPFC was manipulated by tDCS. Furthermore, rTPJ is a key node within the ‘social brain’ for decision-making involved in self-centered and other-regarding concerns (Soutschek *et al.*, 2016; Tang *et al.*, 2017), which has been implicated in sophisticated representations of others’ mental states and integrating these into social decisions (Lockwood *et al.*, 2019). We did not find any modulation effect of stimulation sites, suggesting that all these regions play an important role in prosocial behaviors. Importantly, these regions are functionally and anatomically well-connected (Kennedy and Adolphs, 2012). Stimulating any node of this ‘social brain’ network may activate the whole circuit and elicit comparable behavioral effects. Our results provide evidence supporting that activity in the ‘social brain’, comprising TPJ, dlPFC and vmPFC, is causally linked to prosocial behaviors.

In addition, meta-regression results showed no significant influence of stimulation parameters such as stimulation duration and current intensity. There are some plausible explanations for these non-significant moderating results. First, most studies used typical stimulating parameters such as 12–20 min stimulation durations and 1 ∼ 2 mA intensities of the current. There may not be enough variances between studies to detect the modulation effects of these parameters. Second, the stimulating parameters (stimulation duration and current intensity) used by the investigators were both able to elicit a transient stimulating effect of tDCS. Finally, the small number of included studies may also limit our ability to detect significant moderating effects because of low statistical power.

The current study suffers from a few limitations. First, our meta-analysis only pooled together the studies that assessed the effects of one single session of tDCS, which resulted from the fact that by far no RCTs have explored the medium- or long-term outcomes of tDCS on prosocial behavior. Future studies should evaluate the long-term outcomes of tDCS. Second, the included participant samples were restricted to healthy adults. It remains unclear whether such effects are generalizable to people with psychiatric conditions such as ADHD (Young, 2005), autism (Fontes-Dutra *et al.*, 2019), and schizophrenia (Dodell-Feder *et al.*, 2015), etc. It should be noted that tDCS may exert stronger effects in patients with psychiatry disorders such as autism and schizophrenia (Lee *et al.*, 2018; Kim *et al.*, 2019). Third, due to the complexity of prosocial behavior, only a limited number of studies were included in each specific type of prosocial behavior. This might contribute to the non-significant modulation effect of behavior type, and for this reason, the moderator analysis results documented in the present study require further investigation. Last, most studies included in the current meta-analysis did not measure individual differences at baselines in emotion or trait tendencies (e.g. social value orientation), we were unable to test whether participants’ characteristics were potential modulator factors in our meta-analysis. Future studies are encouraged to systematically examine the role of participants’ characteristics in the tDCS effects on prosocial behaviors. tDCS has become increasingly recognized as a promising tool in neuroscience research for understanding the relationship between brain and behavior in both healthy humans and clinical populations (Filmer *et al.*, 2014). Indeed, several studies have provided converging evidence showing that tDCS is suited to modulate basic cognitive (Kuo and Nitsche, 2012, 2015; Kadosh, 2015) and sensory–perceptual functioning (Costa *et al.*, 2015) and to ameliorate symptoms of many neurological and psychiatric disorders (Brunoni *et al.*, 2012). However, there is still some controversy regarding whether tDCS can effectively change the prosocial behaviors due to the wide range of prosocial behaviors and the heterogeneity in experimental tasks. No previous meta-analysis has systematically examined the effects of tDCS on prosocial behavior. Our meta-analytic study improved our understanding of how prosocial behaviors are linked to the activity of the ‘social brain’ and supported the promising potential of tDCS in modulating high-order social functioning. Our results, for the first time, showed that tDCS effects were not modulated by the types of prosocial behavior, suggesting that tDCS stimulation can be used to improve different types of prosocial behavior and may be effective in treating psychiatric disorders that are characterized by deficits in general social functions. The convergent evidence from the meta-analysis is important to allow valid and reliable interpretation of findings in neurotypical cohorts, but also to allow tailored tDCS protocols to atypical groups with social difficulties.

## Conclusion

Although tDCS has been widely used to change cognition and motor control (Miniussi *et al.*, 2013), the application of tDCS to alter high-level social behaviors is still under development. Our findings point out that both anodal and cathodal tDCS have significant effects on prosocial behaviors, suggesting a causal role of several key nodes within the ‘social brain’ in orchestrating human social behaviors. Given the complexity of prosocial behavior, future research is encouraged to systematically vary the stimulation parameters (e.g. stimulation protocol, current intensity and electrode montage) to gain a better understanding of the beneficial effects of tDCS on social behaviors.

## Supplementary Material

nsab067_Supp

## Footnotes

1 Although both empathy and ToM are indirectly related to prosocial behaviors, we included them in our meta-analysis, because these socio-cognitive abilities are basic of prosocial behavior. In addition, we also reported the anodal and cathodal tDCS effects on prosocial behaviors after removing the empathy and ToM items.

2 It is noteworthy that although the current density was found to significantly moderate the anodal effects tDCS on prosocial behaviors (*Q*_B_(1) = 3.39, *P** *= 0.047), this effect was not replicated in the cathodal tDCS effects. These results suggested that the moderating effect did not exist.
