# Supplementary material for: tDCS effect on prosocial behavior: a meta-analytic review
Source: Soc Cogn Affect Neurosci. 2021 Jun 19;17(1):26–42. doi: 10.1093/scan/nsab067 (PMC8824678; doi:10.1093/scan/nsab067)
Supplement: nsab067_Supp [file nsab067_supp.zip › suppl.docx]

**Supplementary**

**Table S1.** Quality assessment results of the included studies using PEDro Scale.

| **Author & Year** | **Item1** | **Item2** | **Item3** | **Item4** | **Item5** | **Item6** | **Item7** | **Item8** | **Item9** | **Item10** | **Item11** | **Score** |
| --- | --- | --- | --- | --- | --- | --- | --- | --- | --- | --- | --- | --- |
| Adenzato, et al., 2017 | yes | no | no | no | yes | yes | no | yes | yes | yes | yes | 6 |
| Adenzato, et al., 2019 | yes | yes | yes | no | yes | yes | no | yes | yes | yes | yes | 8 |
| Chen, et al., 2019 | yes | yes | yes | no | yes | no | no | yes | yes | yes | yes | 7 |
| Colzato, et al., 2015 | yes | yes | yes | no | yes | yes | yes | yes | yes | yes | yes | 9 |
| Coll, et al., 2017 | yes | yes | no | no | yes | yes | no | yes | yes | yes | yes | 7 |
| Gallo, et al., 2018 | yes | yes | no | no | yes | yes | no | yes | yes | yes | yes | 7 |
| Gross, et al., 2018 | yes | yes | yes | no | yes | yes | yes | yes | yes | yes | yes | 9 |
| Jospe et al., 2020 | yes | yes | yes | no | yes | no | no | yes | yes | yes | yes | 7 |
| Li, et al., 2018 | yes | yes | yes | no | yes | yes | no | yes | yes | yes | yes | 8 |
| Liao, et al., 2018 | yes | yes | no | no | yes | no | no | yes | yes | yes | yes | 6 |
| Liu, et al., 2019 | yes | yes | yes | no | yes | no | no | yes | yes | yes | yes | 7 |
| Luo, et al., 2017 | yes | yes | yes | no | yes | yes | no | yes | yes | yes | yes | 8 |
| Mai, et al., 2016 | yes | yes | yes | no | yes | yes | no | yes | yes | yes | yes | 8 |
| Maréchal, et al., 2017 | yes | yes | no | no | yes | yes | no | yes | yes | yes | yes | 7 |
| Nihonsugi, et al., 2015 | yes | yes | no | no | yes | yes | no | yes | yes | no | yes | 6 |
| Peled-Avron et al., 2019 | yes | yes | yes | no | yes | no | no | yes | yes | yes | yes | 7 |
| Rêgo, et al., 2015 | yes | yes | yes | no | yes | yes | no | yes | yes | yes | yes | 8 |
| Ruff, et al., 2013 | yes | yes | yes | no | yes | yes | no | yes | yes | yes | yes | 8 |
| Santiesteban, et al., 2012 | yes | yes | no | no | no | no | no | yes | yes | yes | yes | 5 |
| Snowdon et al., 2017 | yes | yes | yes | yes | yes | no | no | yes | yes | yes | yes | 8 |
| Tang, et al., 2018 | yes | yes | yes | no | yes | yes | no | yes | yes | yes | yes | 8 |
| Wang, et al., 2014 | yes | yes | no | no | yes | yes | no | yes | yes | yes | yes | 7 |
| Wang, et al., 2016 | yes | yes | no | no | yes | yes | no | yes | yes | yes | yes | 7 |
| Wang, et al., 2020 | yes | yes | yes | no | yes | no | no | yes | yes | yes | yes | 7 |
| Wu, et al., 2018 | yes | yes | yes | no | yes | no | no | yes | yes | yes | yes | 7 |
| Yuan, et al., 2017 | yes | yes | yes | no | yes | yes | no | yes | yes | yes | yes | 8 |
| Zheng, et al., 2017, exp2 | yes | no | yes | no | yes | yes | no | yes | yes | yes | yes | 8 |
| Zheng, et al., 2017, exp1 | yes | no | yes | no | yes | yes | no | yes | yes | yes | yes | 8 |
| Zheng, et al., 2016, exp1 | yes | yes | no | no | yes | yes | no | yes | yes | yes | yes | 8 |
| Zheng, et al., 2016, exp2 | yes | yes | no | no | yes | yes | no | yes | yes | yes | yes | 8 |
| Zinchenko et al., 2019 | yes | yes | yes | no | yes | no | no | yes | yes | yes | yes | 7 |

*Note:* Itme1 = *Eligibility criteria specified*, Itme2 = *Random allocation*, Itme3 = *Concealed allocation*, Itme4 = *Groups similar at baseline*, Itme5 = *Subject blinding*, Itme6 = *Therapist blinding*, Itme7 = *Assessor blinding*, Itme8 = *Less than 15% dropouts*, Itme9 = *Intention-to-treat analysis*, Itme10 = *Between-group statistical comparisons*, Itme11 = *Point measures and variability*.

Fig. S1. An illustration of the tDCS target brain regions. vmPFC *= ventromedial prefrontal cortex;* dLPFC *= dorsolateral prefrontal cortex;* rTPJ *= right temporo-parietal junction.*

Fig. S2. The main experimental paradigms used in the meta-analysis research. Figures were adapted from Robson et al., 2019 and Wang et al., 2014.
