# Supplementary figures and images for: tDCS effect on prosocial behavior: a meta-analytic review
Source: Soc Cogn Affect Neurosci. 2021 Jun 19;17(1):26–42. doi: 10.1093/scan/nsab067 (PMC8824678; doi:10.1093/scan/nsab067)

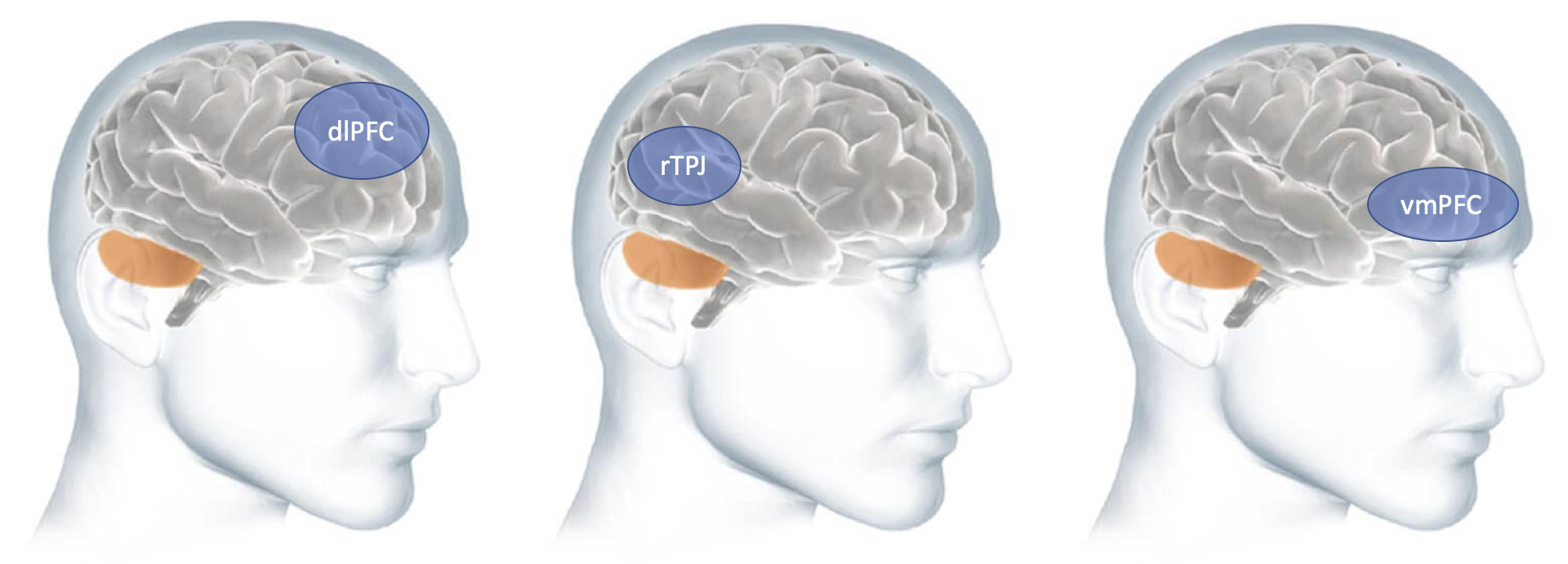

Supplement: nsab067_Supp [file nsab067_supp.zip › scan-20-344-File011.tiff]

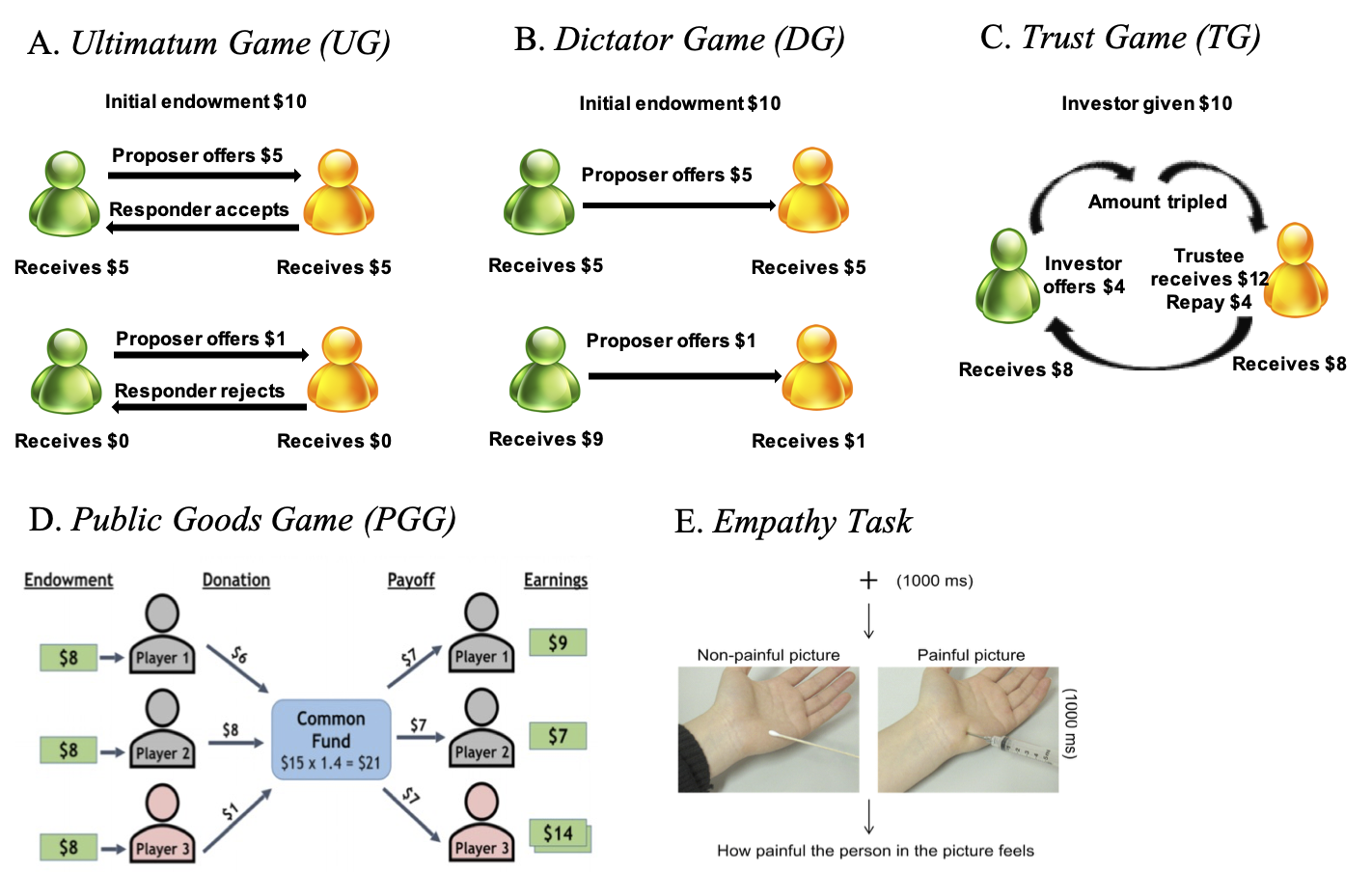

Supplement: nsab067_Supp [file nsab067_supp.zip › scan-20-344-File012.tiff]
